# Cognitive programs: software for attention's executive

**DOI:** 10.3389/fpsyg.2014.01260

**Published:** 2014-11-25

**Authors:** John K. Tsotsos, Wouter Kruijne

**Affiliations:** ^1^Department of Electrical Engineering and Computer Science and Centre for Vision Research, York UniversityToronto, ON, Canada; ^2^Department of Cognitive Psychology, Vrije UniversiteitAmsterdam, Netherlands

**Keywords:** visual attention, executive, visual routines, working memory, selective tuning

## Abstract

What are the computational tasks that an executive controller for visual attention must solve? This question is posed in the context of the Selective Tuning model of attention. The range of required computations go beyond top-down bias signals or region-of-interest determinations, and must deal with overt and covert fixations, process timing and synchronization, information routing, memory, matching control to task, spatial localization, priming, and coordination of bottom-up with top-down information. During task execution, results must be monitored to ensure the expected results. This description includes the kinds of elements that are common in the control of any kind of complex machine or system. We seek a mechanistic integration of the above, in other words, algorithms that accomplish control. Such algorithms operate on representations, transforming a representation of one kind into another, which then forms the input to yet another algorithm. Cognitive Programs (CPs) are hypothesized to capture exactly such representational transformations via stepwise sequences of operations. CPs, an updated and modernized offspring of Ullman's Visual Routines, impose an algorithmic structure to the set of attentional functions and play a role in the overall shaping of attentional modulation of the visual system so that it provides its best performance. This requires that we consider the visual system as a dynamic, yet general-purpose processor tuned to the task and input of the moment. This differs dramatically from the almost universal cognitive and computational views, which regard vision as a passively observing module to which simple questions about percepts can be posed, regardless of task. Differing from Visual Routines, CPs explicitly involve the critical elements of Visual Task Executive (vTE), Visual Attention Executive (vAE), and Visual Working Memory (vWM). Cognitive Programs provide the software that directs the actions of the Selective Tuning model of visual attention.

## Introduction

The corpus of theories and models of visual attention has grown rapidly over the past two decades (see Itti et al., 2005; Rothenstein and Tsotsos, 2008; Nobre and Kastner, 2013). It has become difficult to keep track of these and even more difficult to compare and contrast them with respect to their effectiveness at explaining known phenomena and predicting new ones. Surprisingly, few have attempted to go beyond the creation of saliency maps or re-creation of single cell response profiles. Larger efforts aimed at connecting visual attention with its executive controller or with real-world tasks such as recognition, motor behavior or visual reasoning, are not common. Such a larger scale effort is precisely our long-term goal and a first step will be proposed. The key question addressed is: What are the computational tasks that an executive controller for visual attention must solve? The answer to this question would play a major role in any cognitive architecture. Unfortunately, the previous literature on large-scale cognitive frameworks does not provide much guidance as can be seen from the following synopsis. A great review can be found in Varma (2011).

Dehaene and Changeux (2011), in an excellent review paper, point out that “Posner (Posner and Snyder, 1975; Posner and Rothbart, 1998) and Shallice (Shallice, 1972, 1988; Norman and Shallice, 1980) first proposed that information is conscious when it is represented in an *executive attention* or *supervisory attentional* system that controls the activities of lower-level sensory-motor routines and is associated with prefrontal cortex. In other words, a chain of sensory, semantic, and motor processors can unfold without our awareness, [….] but conscious perception seems needed for the flexible control of their execution, such as their onset, termination, inhibition, repetition, or serial chaining.” This viewpoint puts our effort squarely on the same path as those that address consciousness, however we will stop short of making this link. Our focus is to develop this supervisor for attention so that it is functionally able to provide a testable implementation that uses real images. It can be considered in larger roles as Dehaene and Changeux suggest but we reserve further discussion on this for future work. It might be that by defining concrete mechanisms for executive attention, contributions to our understanding of consciousness will emerge.

Two of the best-known cognitive architectures are SOAR (Laird et al., 1987) and ACT-R (Anderson and Lebiere, 1998). Within SOAR, designed to provide the underlying structure that would enable a system to perform the full range of cognitive tasks, an attentional component was defined named NOVA (Wiesmeyer and Laird, 1990). Attention is claimed to precede identification, is a deliberate act mediated by an ATTEND operator, and functions as a gradient-based, zoom lens of oval shape that separates figure from ground. Attended features move on to recognition. This reflects an “early selection” conceptualization (Broadbent, 1958). ACT-R, designed with the same goals as SOAR, defines perceptual-motor modules that take care of the interface with the environment. Perception operates in a purely bottom-up manner and is assumed to have the function of parsing the visual scene into objects and their features. Attention then is used to select objects and recognize them in a manner that combines a spotlight model with search guidance. The firing of production rules controls shifts of attention. This model also reflects an early selection strategy.

More recently, massive neuronal network simulations have become possible not in small part due to increased computing power and large engineering feats. Zylberberg et al. (2010) develop a large-scale neural system that embodies attention in the form of a router whose job is to set up the precise mapping between sensory stimuli and motor representations, capable of flexibly interconnecting processors and rapidly changing its configuration from one task to another. This captures the information routing part of the problem, but does not include the dynamic nature of attentive single neuron modulations. Eliasmith et al. (2012) describe another large-scale neural model, impressive for its ability to generalize performance across several tasks. The entire vision component is modeled using a Restricted Boltzmann Machine as an auto-encoder, but attention is not used. The major brain areas included in the model are modeled using abstract functional characterizations and are structured in a feed-forward processing pipeline for the most part. Each of these, and in fact most major proposals, view the visual system as a passively observing, data-driven classifier of some sort, exactly the kind of computational system that Marr had envisioned (Marr, 1982) but not of the kind indicated by modern neurobiology. Specifically, the enormous extent of inter-connectivity and feedback connections within the brain (Markov et al., 2014) seem to elude modeling attempts and their function remains a major unknown.

On the neuroscience side, there have been three recent attempts to capture the essence of top-down control for visual attention; executive control has been of interest for some time (e.g., Yantis, 1998; Corbetta and Shulman, 2002; Rossi et al., 2009, and many others). They also provide steps toward understanding the role of the feedback connections. In Baluch and Itti (2011), a map of brain areas and their top-down attentive connections is presented. They nicely overview a number of attentional mechanisms but it is odd that no top-down attentional influences are included for areas V1, V2, and LGN, areas where attentional modulation has been observed (e.g., O'Connor et al., 2002). By contrast, Miller and Buschman (2013) describe several pathways for top-down attention, all originating in frontal cortex and influencing areas LIP, MT, V4, V2, V1. They provide a good picture of top-down attentional connections but not much on exactly how this influence is determined and executed. Finally, Raffone et al. (2014) take an additional important step by defining a visual attentional workspace consisting of areas FEF, LIP and the pulvinar, this workspace being supported by a global workspace in LPFC. This more complex structure likely comes closest to what we seek too, but Raffone et al. do not provide mechanistic explanations as to how this concert of areas operate in a coordinated fashion. Such work subscribes to the philosophy that by combining experimental observations, one can develop an understanding without detailing workable mechanisms. Our perspective is exactly the opposite: we will proceed by trying to determine what needs to be solved first (see Marr's computational level of analysis Marr, 1982) and how those solutions may come about (Marr's algorithmic and representational level). Experimental observations play the role of constraining the set of possible solutions (see Tsotsos, 2014).

Nevertheless, the past work reviewed above has value for our efforts. We notice that each of the above works embody the idea that there exists a sequence of representational transformations required to take an input stimulus and transform it into a representation of objects, events or features that are in the right form to enable solution of a task. This concept is key and points us to Ullman's Visual Routines (VRs). Ullman presented a strategy for how human vision might extract shape and spatial relations (Ullman, 1984). Its key elements included:

- VRs compute spatial properties and relations from *base representations* to produce *incremental representations*,
- base representations are derived automatically, are assumed correct, and describe local image properties following Marr's 2.5D Sketch (1982),
- VRs are assembled from *elemental operations*,
- elemental operations are: shift of processing focus, indexing, boundary tracing, marking, and bounded activation,
- *universal routines* operate in the absence of prior knowledge whereas other routines operate with prior knowledge,
- mechanisms are required for sequencing elemental operations and selecting the locations at which VRs are applied,
- attentive operations are critical and based on Koch and Ullman (1985).
- new routines can be assembled to meet processing goals,
- a VR can be applied to different spatial locations,
- VRs can be applied to both base and incremental representations.

Why is the visual routine a useful concept? The key requirement for a solution to our goal is an approach that is centered on the visual representations important for the completion of perceptual tasks, and transformations between representations that traverse the path from task specification to stimulus presentation to task completion. VRs depend on visual representations and represent algorithms for how one representation is transformed to another toward the overall task satisfaction and as such present us with a starting point for our goal. We will generalize this concept beyond its utility for shape and spatial relation computation, but first look at previous developments of VRs.

A number of researchers have pursued the visual routines concept. Johnson (1993) and McCallum (1996) looked into how VRs may be learned, using genetic programming and reinforcement learning. Horswill (1995) developed a system that performs visual search to answer queries in a blocks world. He included a set of task-specific weights to compute a saliency map, a set of markers that hold the centroids of regions, and a return inhibition map that masks out regions that should not be selected. Brunnström et al. (1996) propose an active approach including an attentional mechanism and selective fixation. They define VRs that can rapidly acquire information to detect, localize and characterize features. Ballard et al. (1997) emphasize the need for an attentive “pointing device” in visual reasoning. Rao's (1998) primitive VR operations are: shift of focus of attention; operations for establishing properties at the focus; location of interest selection. These enable VRs for many visuospatial tasks. Ballard and Hayhoe (2009) describe a gaze control model for event sequence recognition. They highlight problems with saliency map methods for task-based gaze control. VRs also found utility in practical domains: control of humanoids (Sprague and Ballard, 2001); autonomous driving (Salgian and Ballard, 1998); natural language interpretation and motor control (Horswill, 1995); control of a robot camera system (Clark and Ferrier, 1988).

Neurobiologists have also embraced VRs. Roelfsema et al. (2000, 2003) and Roelfsema (2005) have provided neurophysiologic support. They discovered neurons in motor cortex selective for movement sequences. They also monitored the progression of a sequence by recording activity of neurons in early visual cortex, associating elemental operations with changes in neuron response. They thus suggested an enhanced set of VRs: visual search, cuing, trace, region filling, association, working memory, suppression, matching, and motor acts. This work forms a nice stepping-stone onto the path we will take.

However, almost everything has changed in our knowledge of vision and attention since Ullman described visual routines in 1984 and this necessitates at least an update of its conceptualization. We know that attention is more complex than region-of-interest selection for gaze change. It also involves top-down priming of early visual computations, feedback processing, imposes a suppressive surround around attended items to ignore background clutter and modulates individual neurons to optimize them for the task at hand both before the stimulus is presented as well as during its perception. Attentive modulation can change the operating characteristics of single neurons virtually everywhere in the visual cortex (see Itti et al., 2005; Carrasco, 2011; Nobre and Kastner, 2013). Moreover, we know the time course of attentive effects differs depending on task; attentional effects are seen *after* Marr (1982) limit of 160 ms. Further we now know there are no independent modules, as Marr believed, because most neurons are sensitive to more than one visual modality/feature. We also know that the feedforward pass of the visual cortex has limits on what can and cannot be processed. It is not the case that this feedforward pass, as Marr had thought, suffices to compute a complete base representation on which any additional reasoning can take place. If anything, that feedforward pass is only the beginning of the act of perception (Tsotsos, 1990, 2011; Tsotsos et al., 2008). The view that is becoming more accepted is that vision is a dynamic process. For example, Di Lollo et al. (2000) conclude that mismatches between the reentrant visual representations and the ongoing lower level activity lead to iterative reentrant processing. Lamme and Roelfsema (2000) provide a more general view of this idea with motivations from neurophysiology. They show the activity of cortical neurons is not determined by this feedforward sweep alone. Horizontal connections within areas, and higher areas providing feedback, result in dynamic changes in tuning. The feedforward sweep rapidly groups feature constellations that are hardwired in the visual brain, and in many cases, recurrent processing is necessary before the features of an object are attentively grouped. Cichy et al. (2014) provide a comprehensive view of object recognition during the first 500 ms of processing showing that early visual representations (while the stimulus is still on) develop over time and are transient while higher level representations (with greater temporal duration than the stimulus) and various categorical distinctions emerge with different and staggered latencies. Rather than being purely stimulus-driven, visual representations interact through recurrent signals to infer meaning (Mur and Kriegeskorte, 2014). As a result, the vision system is far more complex than Ullman had considered and the control issues become critical.

## What do cognitive programs have to control?

Our original question was “What are the computational tasks that an executive controller for visual attention must solve?” and we posed it in the context of the Selective Tuning (ST) model. ST functionality includes not only the often seen top-down bias signals or region-of-interest determinations, but also overt and covert fixation change, parameter determinations, information routing, localization, priming, and coordination of bottom-up with top-down information. Elements that seem necessary but not currently within ST include representations of task, short-term memory, and task execution. During task execution, results must be monitored to ensure the expected results are obtained. Similar elements are common in the control of any kind of complex system and typically, such tasks are represented within algorithms designed to accomplish control. Such algorithms operate on representations, transforming a representation of one kind into another, which then forms the input to yet another algorithm. Cognitive Programs are hypothesized to capture exactly such representational transformations via stepwise sequences of operations. Cognitive Programs (CPs), an updated and modernized offspring of Ullman's seminal Visual Routines, provide an algorithmic structure to the set of attentional functions and play a role in the overall shaping of attentional modulation of the visual system so that it provides its best performance. We consider the visual system as a dynamic, yet general-purpose processor, tuned to the task and input of the moment. This differs dramatically from what is most common in previous theories of cognition and current computational vision, which regard vision as a passively observing module to which simple questions about percepts can be posed, with the tacit assumption that this suffices for any task. Just and Varma (2007) make exactly the same point after reviewing how recent brain imaging results impact the design of complex cognitive systems. It is important to note that for the balance of this presentation, the motivation for the components of Cognitive Programs arises exclusively from the functional needs of the ST attentional process in its expanded role of tuning the visual system for a given task.

First, let us make the notion of a Cognitive Program more concrete. Ullman defined his visual routines as sequences of elemental operations as described earlier in this paper, and he distinguished universal routines from “regular” ones. Here, Cognitive Programs will be of two types also, but the similarity ends there. The first type is termed *methods*, and whereas Ullman suggested that universal routines can be usefully applied to any scene to provide some initial analysis, and transform input into a representation that is then amenable to the regular kind of routine, here methods cannot be applied without some degree of adaptation to task and/or input (including sensor) characteristics of the moment. For example, a CP method that encodes how to perform visual search needs a specification of the target being sought. It might also be tuned to overall light levels, any context information available, and so on, all useful information for tuning the attentive behavior of the system.

*Scripts* are the executable versions of tuned methods and can be used directly to provide the necessary information to initiate, tune, and control visual processing. Here, all CPs do more than transform one representation into another. They may also encode decision-making elements and set control signals in addition to sequencing representational transformations. The elemental operations differ from Ullman's VRs as well. CPs are composed of accesses to memory (both read and write), yes-no decision points decided by the execution of particular functions, and determination of control signal settings. CPs can be formed by the composition of other CPs. Whereas Ullman's VRs included high-level actions such as boundary tracing as elemental operations, here, tracing will be composed of more primitive elements and will result in a CP of its own. This is simply one example of how CPs may be considered as a more fine-grained version of VRs.

**Figure 1:**
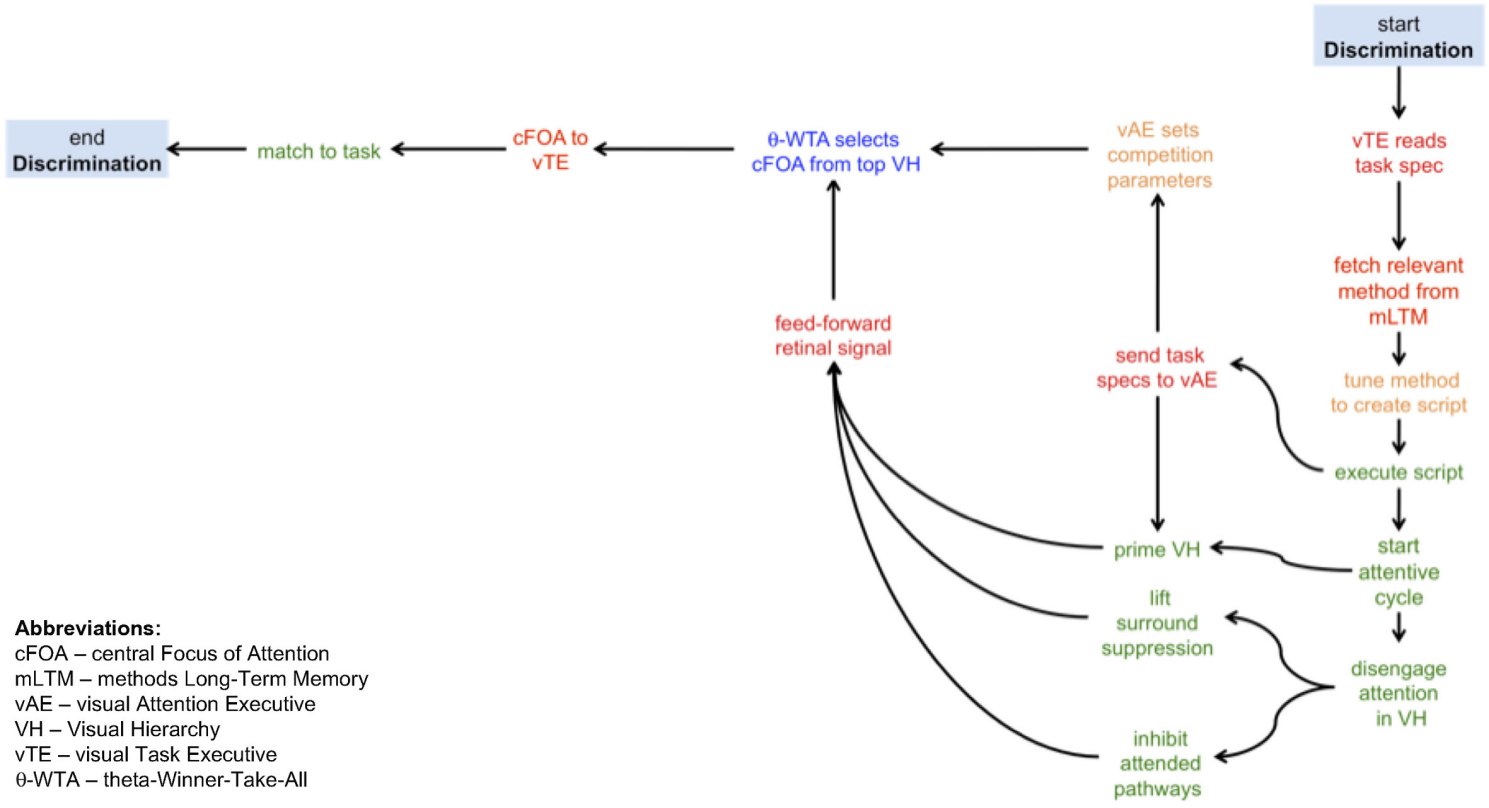
**A graphical depiction of the cognitive program for a visual discrimination task**. The traversal of the graph from start to end provides the algorithm required to execute a discrimination task. The kinds of operations involve several instances of moving information from one place to another (in red), executing a process (in green), making a selection (in blue), or setting parameters (in orange). In words, this algorithm has the following steps: (i) the visual task executive receives the task specifications; (ii) using those specifications, the relevant methods are retrieved from the methods longe-term memory; (iii) the most appropriate method is chosen and tuned into an exectuable script; (iv) the script is then executed, first activating in parallel the communication of the task information to the visual attention exectutive and initiating the attentive cycle; (v) the visual hierarchy is primed using task information (where possible) and in parallel attention is disengaged from the previous focus; (vi) the visual attention executive sets the parameters for executing the competition for selecting the focus of attention; (vii) disengage attention involves inhibiting the previously attended pathways and any previously applied surround suppression is also lifted (note that steps v–vii are executed in parallel before the visual stimulus appears, to the extent possible); (viii) the stimulus flows through the tuned visual hierarchy in a feed-forward manner; (ix) the central focus of attention is selected at the top of the visual hierarchy; the central focus of attention is communicated to the visual task exectuive that then matches it to the task requirements; (x) if the selected focus and the task requirements match, the task is complete.

A sample CP may help clarify their form. Figure 1 shows a simple CP method, one intended for the visual task of discrimination. Acronyms and some components are not fully defined until a subsequent section; this example is given in order to show only the form of CPs and the kinds of elemental operations that will come into play. Discrimination, following Macmillan and Creelman (2005) is defined as a task where a yes-no response is required on viewing a display with a stimulus drawn from one of two classes, and where one class may be noise. As can be seen from the figure, the kinds of operations involve several instances of moving information from one place to another (in red), executing a process (in green), making a selection (in blue), or setting parameters (in orange). The first step is for the visual task executive (vTE) to receive the specification of the task (from an unspecified source external to this model). The details of the task can be used as indices into the methods long-term memory (mLTM) in order to select and fetch the most appropriate method. This implies that the memory itself is organized in an associative manner that reflects key task elements. The chosen method is then tuned using the task specification and becomes an executable script. The script initiates the attentive cycle, and sends the elements of the task that are required for attentive tuning to the visual attention executive (vAE). The vAE then primes the visual hierarchy (VH) with the appropriate top-down signals that reflect expectations of the stimulus (e.g., the display will consist of a ring of 8 items) or instructions to the subject (e.g., search for the green item) and also sets any parameters needed for stimulus competition for attention. How is it possible to communicate a “cue” to a subject? One way is to simply show the cue; it would be processed by exactly the same system, attended, and the resulting output representation (later termed *attentional sample*) used as the basis for priming the system for the upcoming stimulus. While priming is occurring, attention is also being disengaged from its previous focus, and here disengage means that any attentive spatial surround suppression (Hopf et al., 2010) or feature surround suppression (Störmer and Alvarez, 2014) imposed for previous stimuli is lifted, and any previously attended pathways are inhibited (implementing an object-based inhibition of return). This last set of functions gives an excellent example of predictions this kind of analysis provides. The notion of disengaging attention is a common one in the literature but it has not previously been operationalized. Here, an operational definition is presented, amenable to experimental verification, and functionally consistent with the needs of ST. Continuing with the example, once all of this is complete, the feedforward signal appears and traverses the tuned VH. In other words, these actions would occur before stimulus onset, consistent with Müller and Rabbitt's (1989) conclusion that in order for priming to be effective subjects must be informed of it 300–80 ms before stimulus onset. Once the feedforward pass is complete, ST's θ-WTA process (a winner-take-all decision process based on a binning threshold θ that selects a spatially contiguous set of largest values within some retinotopic representation, such as the responses of a specific neural selectivity or filter across the visual field—see Tsotsos et al., 1995; Rothenstein and Tsotsos, 2014) makes a decision as to what to attend and passes this choice on to the next stage. The vTE, which is monitoring the execution of the script, then takes this choice, compares it to the task goals, and decides on whether the discrimination task is completed in a positive or negative manner and the task is complete.

It may seem that a neural realization of such an abstract and complex process is doubtful. However, recently, Womelsdorf et al. (2014) have detailed a broad variety of simple neural circuit elements that provide precisely the kinds of functionality CP's require, including gating, gain control, feedback inhibition and integration functions. An important future activity is to see how to assemble such circuit elements into the functions described here for CPs.

Now it is clear how CP's are the software for the vision executive; the example of Figure 1 is a *flowchart* representing the algorithm that may solve discrimination. There is no illusion here that this specification is all that is needed. Much additional processing is required by each of the components, but the additional computations are all known and fit into the existing ST methodology. However, at an abstract level, this description suffices. In comparison with Ullman's visual routines, this description has a finer grain of detail.

A brief overview of ST is in order. For a full description of ST see Tsotsos (2011), Rothenstein and Tsotsos (2014)—those details will not be repeated here. The roots of ST lie in a set of formal proofs regarding the difficulty of comparing one image to another using the methods of computational complexity (Tsotsos, 1989, 1990). It was shown that a passive, feedforward pass was insufficient to solve the task in its general form, given the resources of the human brain. This paradox—the human brain is very good at solving this problem—underlies the implausibility of vision as a passive observer and points to a dynamically modifiable, active vision process. Further, the range of visual tasks humans perform require a time course often longer than that provided by a single pass through the visual cortex. This characteristic, with the flexibility to tune or parameterize, functionally re-purposing the processing network for each pass, distinguishes ST from its competitors (recurrence in a dynamical system or in a neural network is not the same).

**Figure 2:**
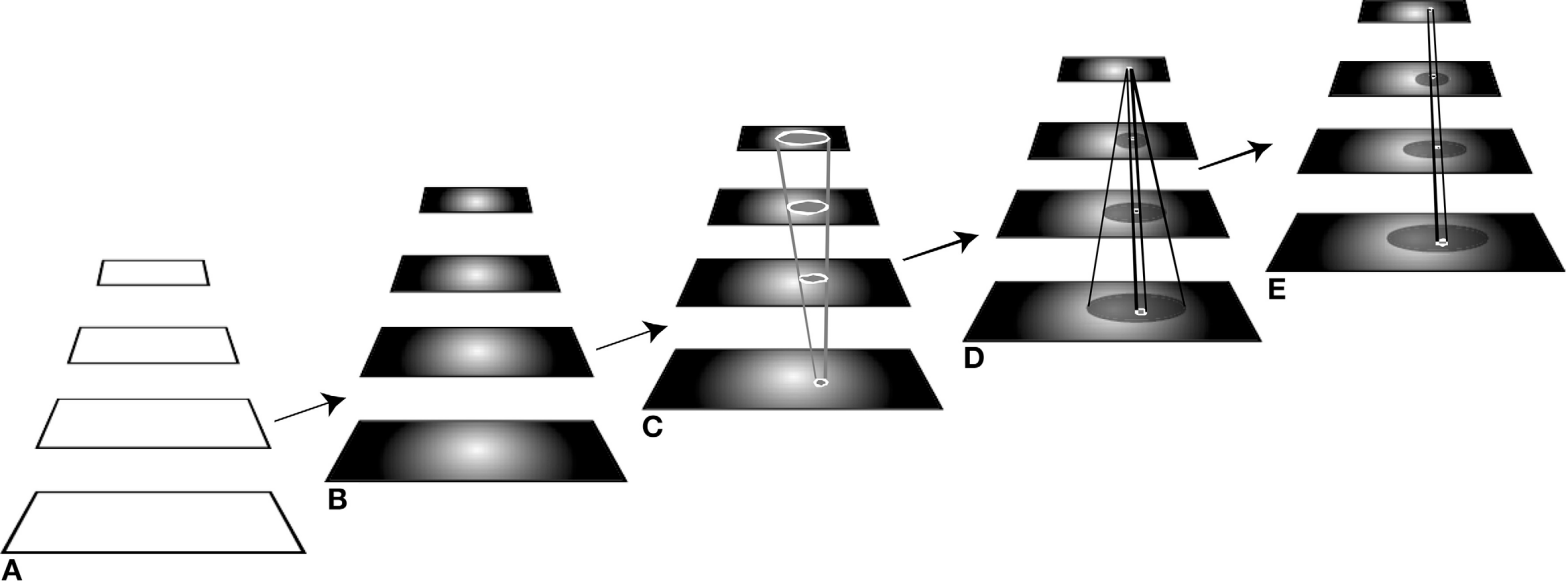
**The different stages of processing of the visual hierarchy needed for different visual tasks**. The five components of the figure represent processing stages ordered in time, from left to right. **(A)** In the first stage, the network is portrayed as “blank,” that is, without stimulus or top-down influences, as it might be prior to the start of an experiment, for example. **(B)** The second stage shows the network affected by a top-down pass tuning the network with any priming information to set up its expectation for a stimulus to appear, when such information is available. Here, the network is set up to expect a stimulus that is centrally located. **(C)** At this point, the stimulus appears and is processed by the tuned network during a single feedforward pass. **(D)** If the required task for this stimulus cannot be satisfied by the first feedforward pass, the recurrent localization algorithm is deployed that traverses the network in a top-down manner, identifying the selected components while suppressing their spatial surrounds. **(E)** A subsequent feedforward pass then permits a re-analysis of the attended stimulus with interfering signals reduced or eliminated. See text for further explanation.

In Figure 2, a caricature of the visual processing hierarchy is shown for descriptive purposes. It is intended that this simple 4-level structure represent the full ventral and dorsal visual networks and from here on, the acronym VH refers to this. The manner in which ST operates on a visual hierarchy shows how feedforward and recurrent traversals are inter-leaved. Within ST, the basic attentive cycle consists of a first stage, labeled B in Figure 2, that represents a task-based priming stage. The time period of this stage can range from 300 to 80 ms before stimulus presentation, as described along with Figure 1. That is, if during an experiment some priming signal is shown to a subject within that time period, there is enough time for it to be processed so that it affects the perception of the test stimulus. Here, not only is this scenario covered, but also any priming can be included, such as the impact of world knowledge; it is assumed that in order to affect processing, a top-down traversal of VH would be required based on the content of the priming stimulus. The next stage, C, is the stimulus-driven feedforward processing stage (requiring about 150 ms for a full traversal), followed by selection and task-specific decision. Then stage D, a recurrent tracing, localization and surround suppression stage (needing about 100–150 ms for a full top-down pass), and E, a modified feedforward processing stage that permits a re-computation of the stimulus with background clutter suppressed with the intent of optimizing neural responses to the attended item.

Each of the stages is parameterized differently depending on task. Some of the stages may not be needed for a given stimulus and task. If the decision stage C, for example, determines that the task is satisfied by the output of the first feedforward stage, then no further stages are needed. For the visual tasks of discrimination, categorization, or identification (in all cases following their definitions in Macmillan and Creelman, 2005; Tsotsos, 2011), stages A–C usually suffice. For the tasks of within-category identification, A–D are needed with the option of stage D requiring only a partial recurrent pass. Full localization tasks require a complete stage D, while segmentation, visual search, and other more complex tasks require all stages and perhaps multiple repetitions of the cycle. These stages are more fully described in Tsotsos et al. (2008), Tsotsos (2011). Any controller will have to manage these differences.

The requirement for an additional top-down pass for localization is not inconsistent with the claims of Isik et al. (2014). There, it is shown that IT neural representations encode position information that can be decoded by a classifier, and thus the authors conclude that position is represented with a latency of about 150 ms, consistent with a feedforward progression through the visual hierarchy. In ST, it is the recurrent localization process that replaces the role of the classifier, and in contrast to current classifiers presents a biologically plausible mechanism (and is partially supported experimentally, Boehler et al., 2009). It also provides a mechanism for tracing down to earlier levels, functionality that classifiers do not possess, and thus providing more detailed position information if required. This highlights the conceptual difference between the time at which information is *available* from which position may be computed—which the Isik et al. paper well documents—and the time at which that information is decoded and made *usable* for processes needing position information, which is what ST can accomplish. Further, a simple classifier cannot easily determine position from a spatial representation containing multiple objects; a selection method is needed, and ST provides this.

**Figure 3:**
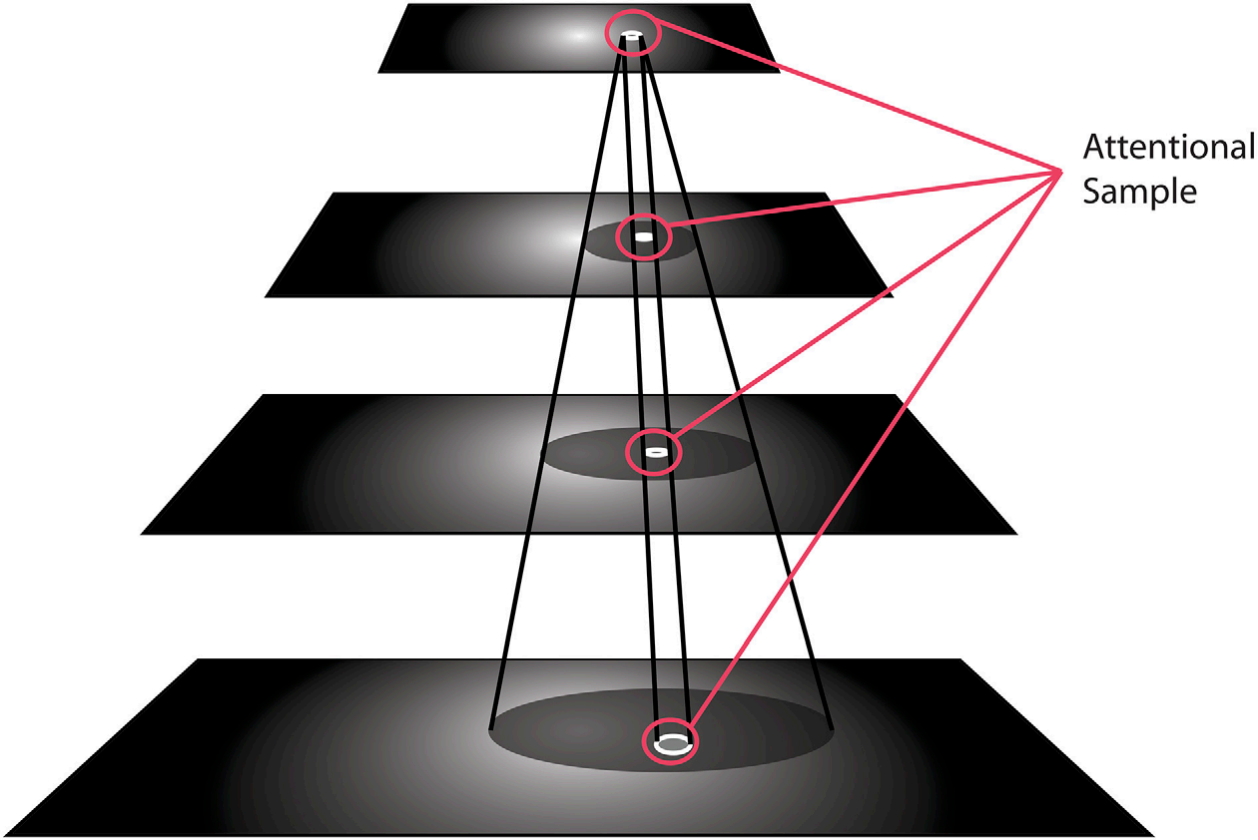
**The visual hierarchy is shown with the elements within each layer selected by ST's recurrent localization process, which together are identified as the Attentional Sample**.

**Figure 4:**
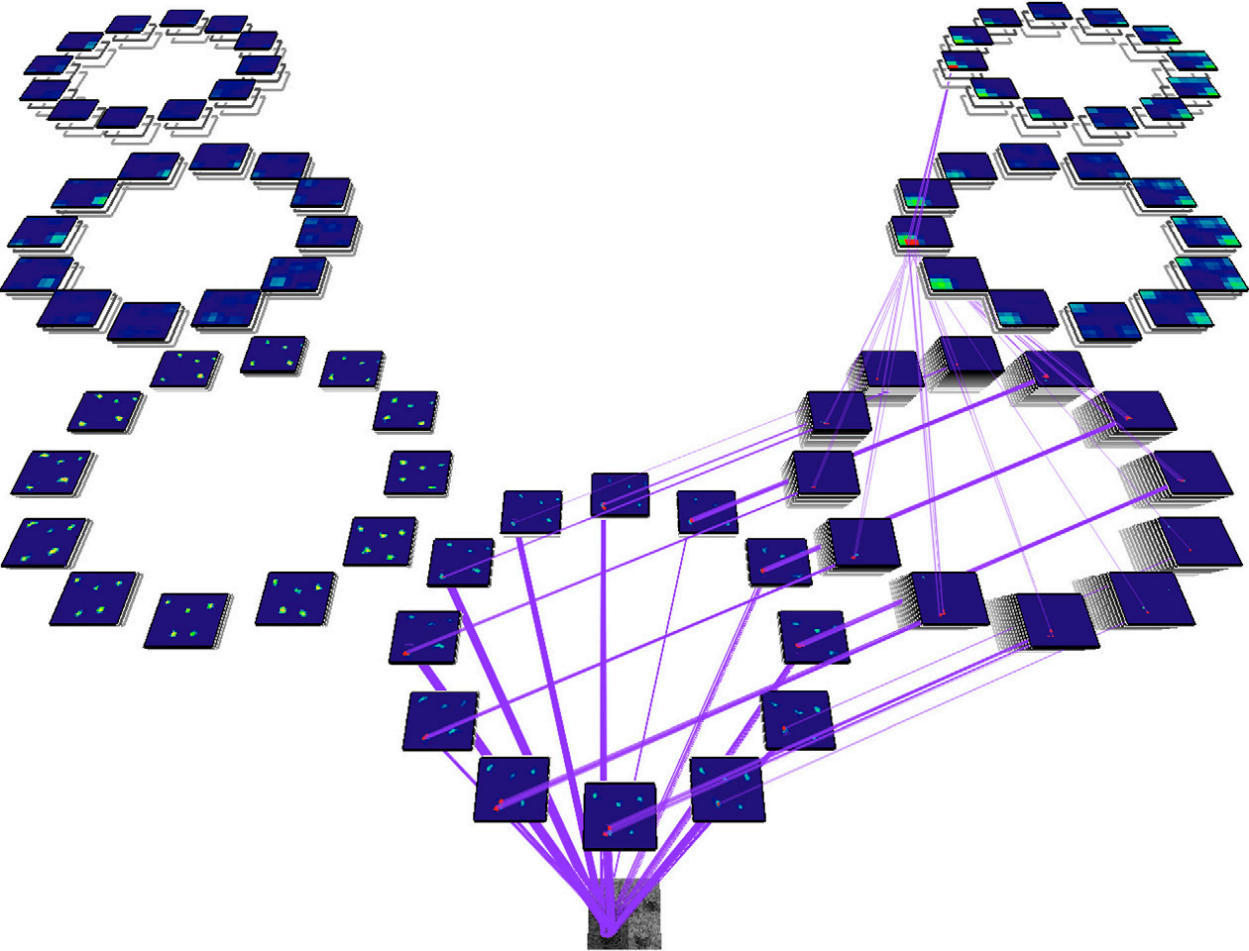
**An actual model output from a real image sequence showing the identification of the attentional sample as the set of intersection points of the purple lines (the neural connections traced by the recurrent localization process) and each of the feature map representations (the blue rectangles each represent one feature filter and within each, the colored areas denote responses of varying strength to input stimuli)**. Full detail on how this example is computed appears in Tsotsos et al. (2005a). Briefly, for the purpose required here, the input image is at the lower end of the diagram, is processed first by a set of filters representing area V1 motion processing, whose output then splits to continue to further processing levels. On the left is the further abstraction of translational computation at coarser spatial resolutions (areas MT, MST, and 7a) while the right hand side is concerned with computation for spatial velocity gradients, spiral motion (rotation, expansion, etc.) and full field egomotion (areas MT, MST, and 7a). In total, there are 654 separate filter types in this hierarchy. The recurrent localization process begins at the top, selects strongest responses, and then refines that selection tracing back the neural inputs that are responsible for that strongest, top-level response (Tsotsos, 2011).

There is one important concept to introduce at this point, namely that of the Attentional Sample (AS), which was mentioned earlier. During the recurrent tracing stage (Figure 2D), θ-WTA decision processes at each level of the hierarchy select the representational elements computed at that level that correspond to the attended stimulus^1^. It will not always suffice to make this selection at the highest level, say at the level of object categories for example. Some tasks will require more details, such as locations of object parts, or feature characteristics. In general, the AS is formally a subset of the full hierarchy, that is, the set of neural pathways from the top of the hierarchy to the earliest level including all intermediate paths, and where at every level there is a connected subset of neurons with spatially adjacent receptive fields that represent the selected stimulus at that level of representation. The recurrent localization process will select these portions of the representation. Multiple stimuli, that are distinguishable from one another on the basis of their constituent features, are not typically selected together as part of a single AS. Since the selection occurs in a top-down fashion, each selection becomes part of the overall attentional sample that represents what is being attended and it can be added to working memory, as will be seen below. Figure 3 provides an illustration of the concept of an attentional sample with its components highlighted on the appropriate processing stage in Figure 2, while Figure 4 shows the AS computed by the functioning model. Figure 4 shows a snapshot of how ST attends to a rotating object in an image sequence. Not only is the object selected as a portion of the input image—this is a common result of most attention models—but ST also connects that input selection to the particular neurons that have played a role in its selection throughout the processing hierarchy. In other words, if each level of processing computes selectivity of a different kind of feature abstraction (velocity, direction, velocity gradient, rotation/expansion/contraction, etc.), this feature set is localized within the hierarchy and can be thought of as the feature vector that best describes what is attended. It is this attentional sample that is then used by other visual computations for further processing. A classifier might consider this AS as its input. To draw a further comparison to Isik et al. (2014), the position information Isik et al. refer to is what is represented at the top level of the hierarchy only. It is position at its coarsest spatial resolution. ST on the other hand, provides not only that but also position at increasingly higher spatial resolutions through the hierarchy, to the level needed by task requirements.

## Extensions of selective tuning to enable cognitive programs

A new functional architecture, based on Selective Tuning, for executive control via a Cognitive Program strategy can now be proposed (and is an extension of Kruijne and Tsotsos, 2011; Tsotsos, 2013). It must be stressed that this architecture was developed not by examining the literature to see what functions are attributed to, for example, working memory, or other functional units. Rather, the only components of function included are those that the algorithm for Selective Tuning requires (e.g., computation of its various parameters or control signals). This is a risky approach because it might seem that there are obvious missing pieces or inconsistencies. However, it is a unique approach in that it uses the ST foundation, which has been proven in many ways and provides a strong functional base, something that purely experimental work does not. In other words, here we present a strategy designed in a top-down manner as required by an existing successful algorithm, with the ultimate goal of trying to discover new components or functions that might stand as predictions for future experimental work. As such, the architecture stands as a hypothesis and there is no claim whatsoever that this functional architecture suffices to explain the existing literature in its full breadth and detail. However, it is claimed that it will suffice to augment ST to enable it to execute a broad family of visual tasks in a manner that is extensible to more complex tasks and is consistent with much (but perhaps not all) of the relevant aspects of human visual performance.

**Figure 5:**
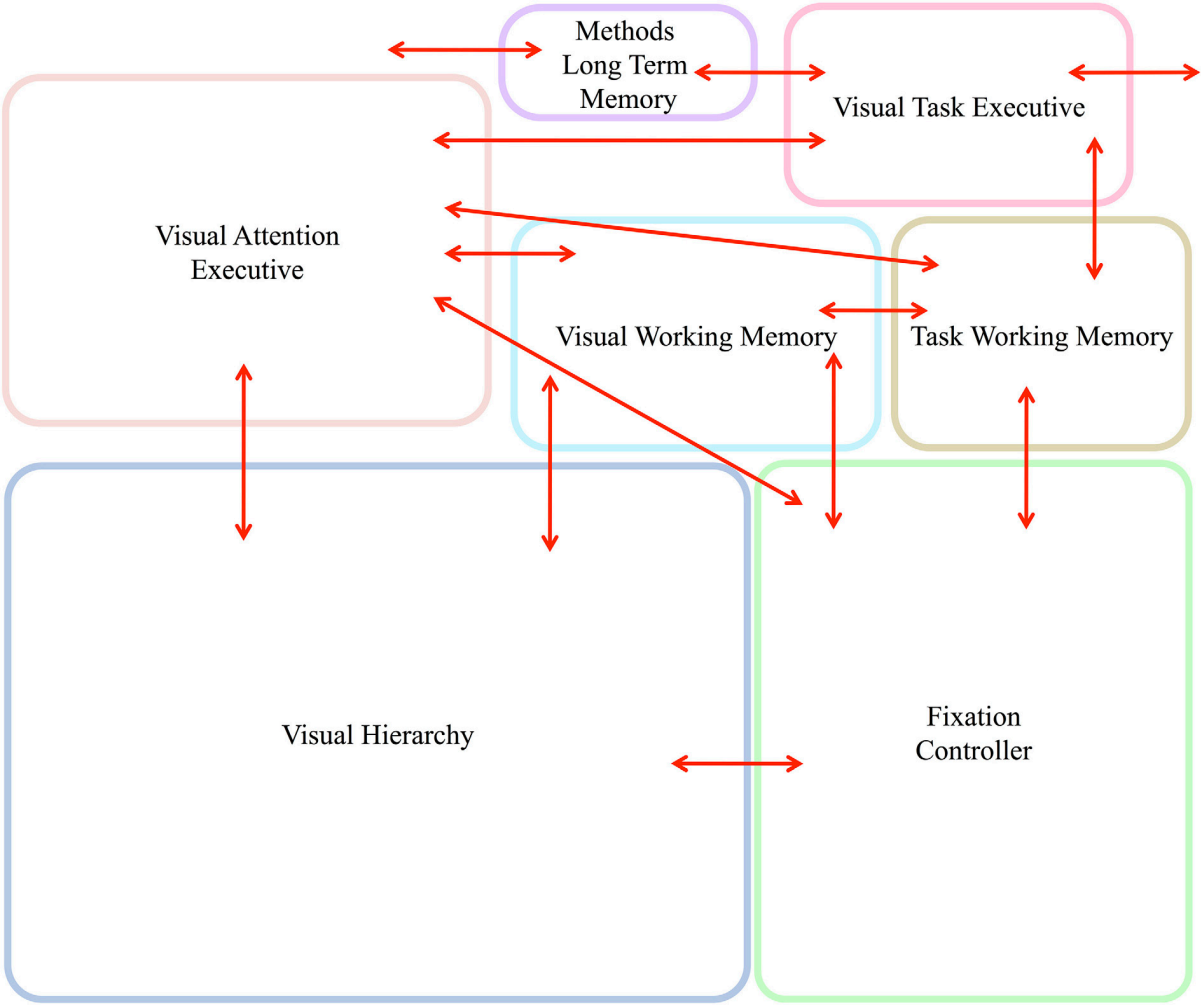
**The abstract, block-diagram, structure of the functional components required to support the executive control of attentive processing with communication channels indicated by the red arrows**. In Figure 6, this block-diagram is further detailed with the internal components for each, using the same block colors. The arrows without endpoints connect to external components.

Figure 5 gives the block diagram of the major components needed and their communication connections. Brief descriptions of each follow. Evidence from primarily human studies as to the functionality of such components is detailed in Kruijne and Tsotsos (2011) and, due to space limits, will not be repeated here.

The visual hierarchy, (VH), in a form that is amenable to the ST method of attention, represents the full ventral and dorsal streams of the visual processing areas. Partial implementations have been previously reported (Tsotsos et al., 2005a; Rodríguez-Sánchez and Tsotsos, 2012). The qualification of “amenable to the ST method of attention” is important. The majority of current popular visual representations such as HMAX (Riesenhuber and Poggio, 1999) or Convolution Nets (LeCun and Bengio, 1995) contain components that make them entirely unsuitable for attentive processing of the kind ST employs, among them feedforward max-pooling operations. Since ST requires a top-down, recurrent max-finding operation, methods that choose maximum responses on the feedforward pass make their decisions too early (and against Marr's principle of least commitment, 1982) and prevent the recurrent method of ST, or perhaps even any recurrent process at all. Arguments as to why ST's recurrent version is more consistent with known neurobiology are provided in Tsotsos (2011).

The Fixation Control mechanism (FC) was first described in Tsotsos et al. (1995) and a cursory implementation was shown. It has since been further detailed and completely implemented (Zaharescu et al., 2005; Tsotsos, 2011; Wloka, 2012), but its details will not be included here. The fixation control mechanism includes two important representations. The first is the Peripheral Priority Map (PPM) that represents the saliency of the peripheral visual field, biased by task and computed using the AIM algorithm (Bruce and Tsotsos, 2009). The other is the History Biased Priority Map (HBPM) which combines the focus of attention derived from the central visual field (cFOA—defined as the image region with strongest response profile at the highest levels of representation within the visual hierarchy) after processing by the full visual hierarchy and the foci of attention derived from the peripheral visual field (pFOA), i.e., the top few most salient items of the PPM. The point is to provide a representation that includes central fixation items (that do not require gaze change), peripheral fixation items (that do require gaze change), and task influence on these, on which computations of next target selection can be performed.

The Long Term Memory for methods (mLTM) stores CP methods, as described earlier. Where CPs might come from is not addressed here; we may assume that they are learned through some unspecified process external to this framework. Figure 1 shows an example method (and was detailed earlier). An important characteristic of mLTM is that is will require a powerful indexing scheme to enable fast search among all of the methods for the particular ones most relevant to the task at hand. That is, in an associative manner, elements of the task description should quickly identify relevant methods.

Visual Working Memory (vWM) contains at least two representations. Within the vWM is the Fixation History Map (FHM) that stores the last several fixation locations. Each decays over time but while active provides the location for location-based inhibition-of-return (IOR) signals. This inhibition is intended to bias against revisiting previously seen locations (Klein, 2000) but can be over-ridden by task demands. The second representation is the Blackboard (BB), introduced in Tsotsos (2011) and where more details can be found. The BB stores the current *attentional sample* (the selected locations, features, concepts at each level of the VH as described earlier) determined by the recurrent attentional localization process so that it may be used by all other components.

Task Working Memory (tWM) includes the Active Script NotePad which itself might have several compartments. One such compartment would store the active scripts with pointers to indicate progress along the sequence. Another might store information relevant to script progress including the sequence of attentional samples and fixation changes as they occur during the process of fulfilling a task. Another might store relevant world knowledge that might be used in executing the CP. The Active Script NotePad would provide the vTE with any information required to monitor task progress or take any corrective actions if task progress is unsatisfactory.

The vTE reads tasks, selects task methods, tunes methods into executable scripts, deploys scripts to tune the vision processes, and monitors and adapts script progress. It receives input in the form of a task encoding from outside the structure of Figure 5. Sub-elements include the Script Constructor that tunes methods into scripts, the Script Executor that moves along the script step by step, sending the appropriate commands to the correct places, and the Script Monitor. The Script Monitor checks each step of the script to ensure the appropriate results are achieved. The full details of task execution are represented by the attentional sample AS, and the sequence of AS's, fixations, and other information stored in the Active Script NotePad. In other words, it has access to the history of important computations performed and their results during the process of performing the task. If those details do not confirm script success, there might be remedial action taken by making small alterations to the script or replacing the current script by a different one.

The vAE contains a Cycle Controller, algorithms to translate task parameters into control signals, and communicates with external elements. The Cycle Controller is responsible for initiating and terminating each stage of the ST process (shown in Figure 2). For example, it would initiate the θ-WTA process for the top of the VH in order to determine the focus of attention in the central visual field (cFOA). The vAE also initiates and monitors the recurrent localization process of Figure 2D. This process is fully detailed in Rothenstein and Tsotsos (2014). There, we present the implementation of ST's neural encoding scheme integrated with attentional selection. We show that it models firing rates observed in experimental work on single cells as well as across hierarchies of neurons. The Cycle Controller cycles repeatedly until the task is complete, a determination that is made by the vTE.

**Figure 6:**
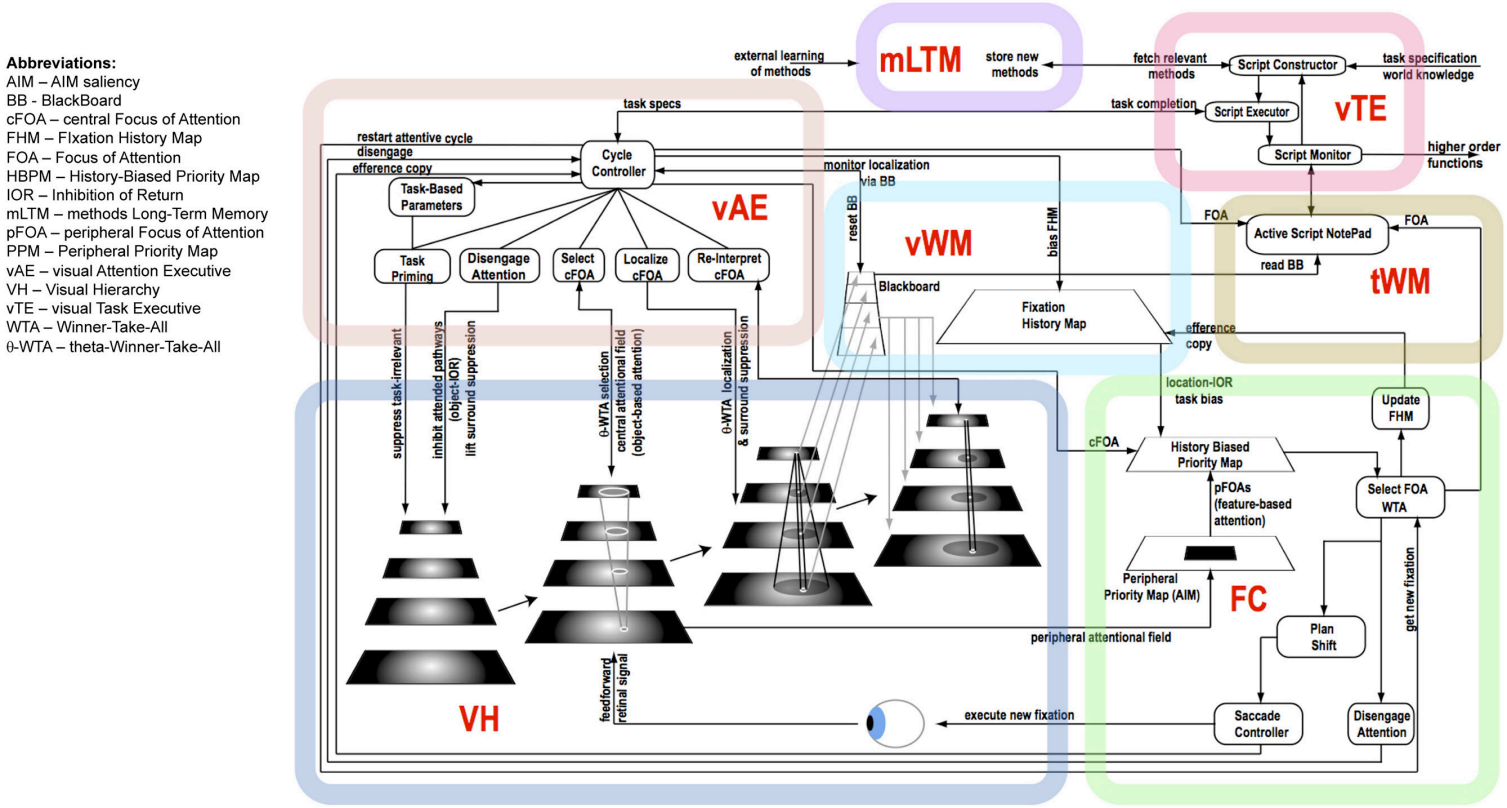
**The details of the architecture and communication pathways required for Cognitive Programs to be interpreted as controlling sequences of instructions for visual task execution**.

Figure 6 shows the details of the major components shown in Figure 5 and graphically links the details of the descriptions above to each other. Importantly, it shows the various control signals and information pathways identified to enable ST to function. The figure highlights several functional components that also stand as experimental predictions, among them:

- there are two kinds of IOR, a location-based IOR and an object-based IOR, each arising from different processes and appearing at different times during an attentive cycle.
- feature-based attention and object-based attention arise from selections on different representations, and thus appear at different times during an attentive cycle.
- the commonly used notion of “disengaging attention” is defined as a particular set of actions, namely, lifting surround suppression, both spatial and featural, on the previous focus of attention, and inhibition of the neural pathways involved in the previous focus of attention (this is object-based IOR).
- computation of saliency is performed only in the periphery (outside 10° or so of visual angle) based on the early representations of the VH.
- selection of feature-based attention foci is based on the Peripheral Priority Map.
- selection of object-based attention foci is based on the central attentional field (central 10° or so) of the highest layers of the VH.

There are more characteristics that stand as experimental predictions. One dimension of prediction that is not included is that of associating specific brain areas to the functions in the figure. Although it is possible to make some associations (for example, VH represents the set of ventral and dorsal visual areas, the BB may be part of the pulvinar, the PPM and HBPM may be area V6, the FHM may be part of FEF—justifications for these appear in Tsotsos, 2011), we refrain from emphasizing these. The reason is simply that it is more important to confirm the function of each component and of the framework as a whole. Once we have strong evidence that the correct functional pieces are included, we can start to consider which brain areas might correspond.

It is important to ensure that there is sufficient justification for the decision to functionally separate the elements described in this section. For example, why separate the tWM from the vWM? Or the vAE from the vTE? In both cases, the intent is clear. The tWM is intended to keep track of any information that relates to status and progress relating to completion of the task at hand. Think of it as the storage for each of the major checkpoints that must be satisfied during task execution, whether due to visual, motor, reasoning or other actions. The vMW, on the other hand, stores all the actual visual information extracted from the input stream and processing by the VH, whether they correspond to components of task checkpoints or not. It corresponds to whatever is seen and remembered for short-term processing and provides input to the determination of whether or not checkpoints are satisfied. The vAE and vTE have a similar distinction. The vAE applies its processing to the VH only; it controls the VH to adapt it to the task and input. The vTE is not concerned with this but focuses on setting up all the task components into an executable script, and of course, this includes the attentional aspects. Functionally it makes some sense to have separate components in both cases, even though it may appear as if it might be possible to embed the vWM within the tWM and the vAE within the vTE. There is little or no empirical evidence of which we are aware for either strategy in the brain; it is clear that from a pure modeling perspective either approach can be made to function correctly. The separation suggested is a logical one and would stand as a prediction for future experimental work.

**Figure 7:**
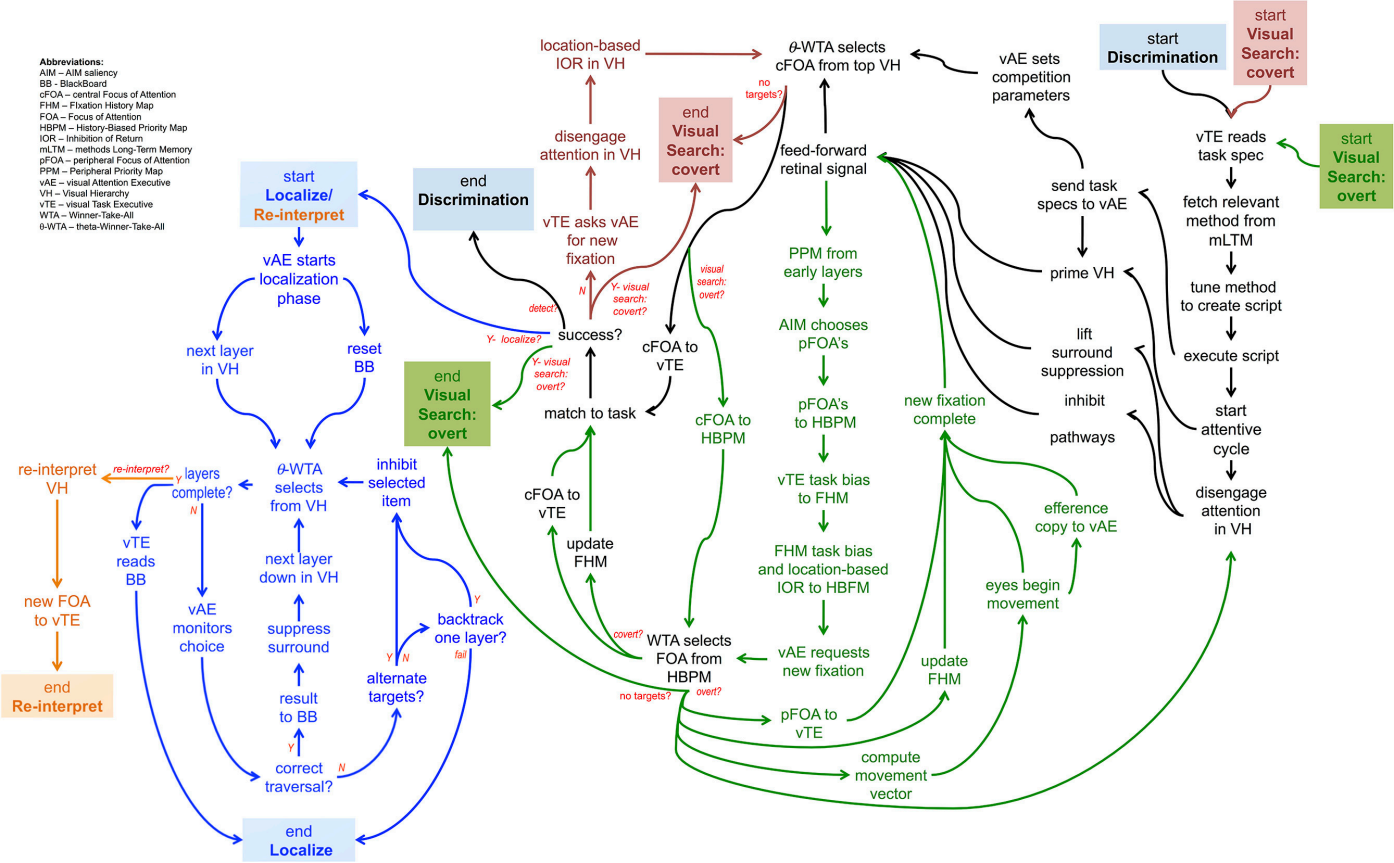
**A graphical depiction of the flow of commands of 5 different CP's, one built upon another, together providing a non-trivial, yet far from complete, description of how some simple visual tasks may be controlled**. Each CP is denoted by its color. Each CP beyond the Discrimination one, involves the composition of more than one CP.

Figure 7 shows a set of linked method CPs, extensions to the Discrimination CP described earlier. The Discrimination CP has been previously described while the Visual Search: Overt CP will be used in the example of the next section. The Visual Search: Covert CP is a straightforward extension of Discrimination while the Localize/Reinterpret CP reflects the recurrent localization mechanism of the descriptions accompanying Figures 2, 3.

## The curve tracing example

We turn now to a simple example, one that has appeared in the context of visual routines previously (Jolicoeur et al., 1986). Their main experimental task was to quickly decide whether two Xs lay on the same curve or on different curves in a visual display. Mean response time for “same” responses increased monotonically with increasing distance along the curve between the Xs. The authors, based on this and similar results on a related experiment, concluded that humans can trace curves in a visual display internally at high speed (the average rate of tracing was about 40° of visual angle per second). The curves were displayed approximately foveally, with the distance between the Xs being between 2.2° and 8.8° of visual angle. There were no cross points of the curves, and it seems the curves were “simple” and not overly close to one another nor convoluted in shape. The authors conclude that curve tracing is a basic visual process. Here, we show how the CP strategy can provide an explanation for curve tracing but in order to make the demonstration more interesting, the display is assumed to be large enough to require eye movements. The same strategy, as should be apparent, can deal with smaller displays as Jolicouer et al. use, that do not require eye fixation changes.

**Figure 8:**
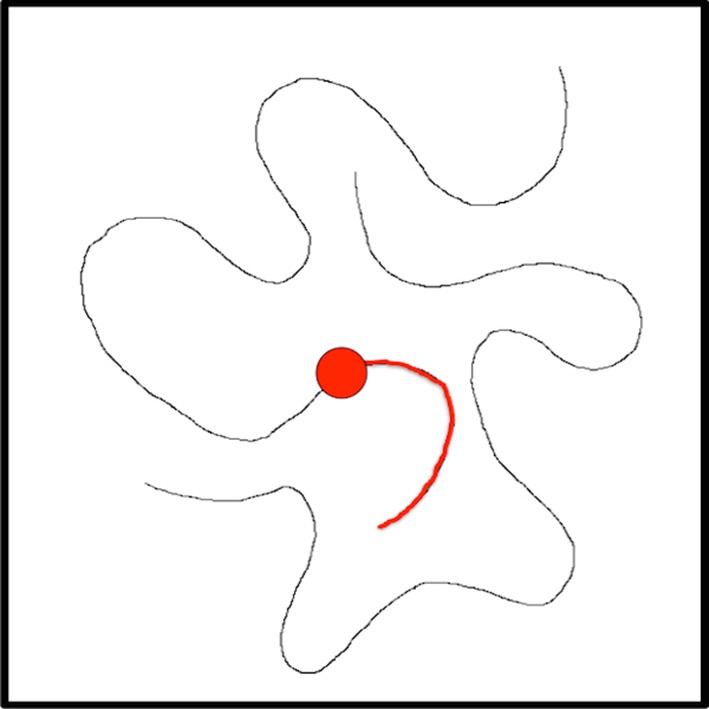
**Suppose the task is to trace this curve**. The current fixation is at the red dot, the visual processing hierarchy has been biased to be more selective to curved lines, and the curve portion highlighted in red has already been tracked and this is recorded in the Active Script NotePad.

**Figure 9:**
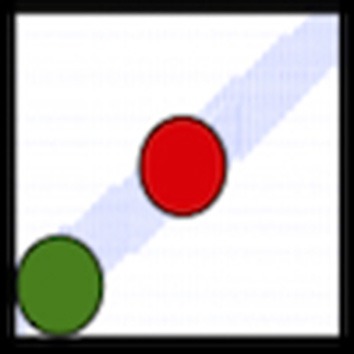
**This figure represents the central attentional field (the central 10° or so of the image) representation at the top of the VH**. The curve at current fixation has already been attended; the attentional sample in BB contains its details. The central field is examined via the θ-WTA mechanism to find largest responding element other than the current fixation; the already tracked curve has been suppressed by the inhibition of return mechanism. The green dot is selected as the central attentional focus.

**Figure 10:**
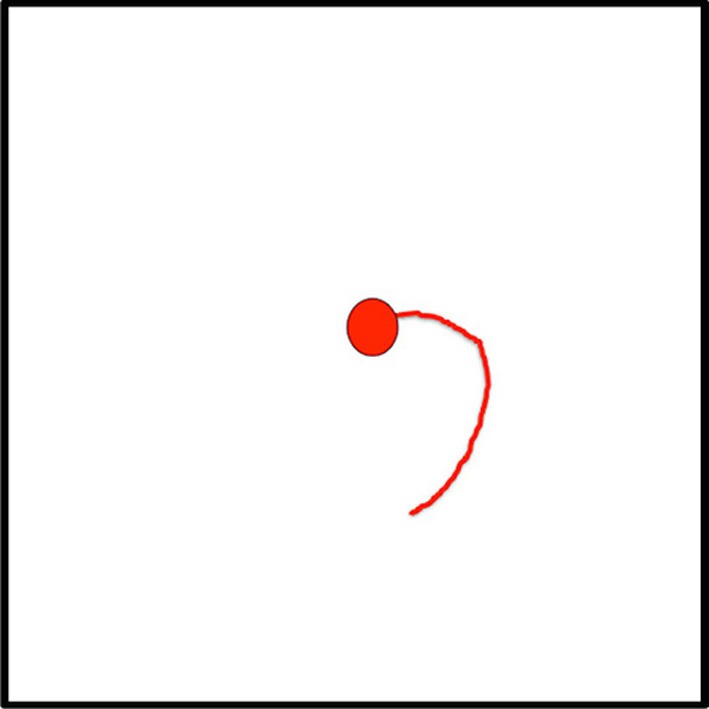
**The figure represents the Fixation History Map**. The FHM represents the previous fixation (red dot) and the already traced portion of the curve in red. These provide the inhibition of return bias for the HBPM. Note how the FHM represents a spatially larger area than the visual field because it must also include extra-retinal space in order to reduce the possibility of incorrect gaze oscillatory behavior.

**Figure 11:**
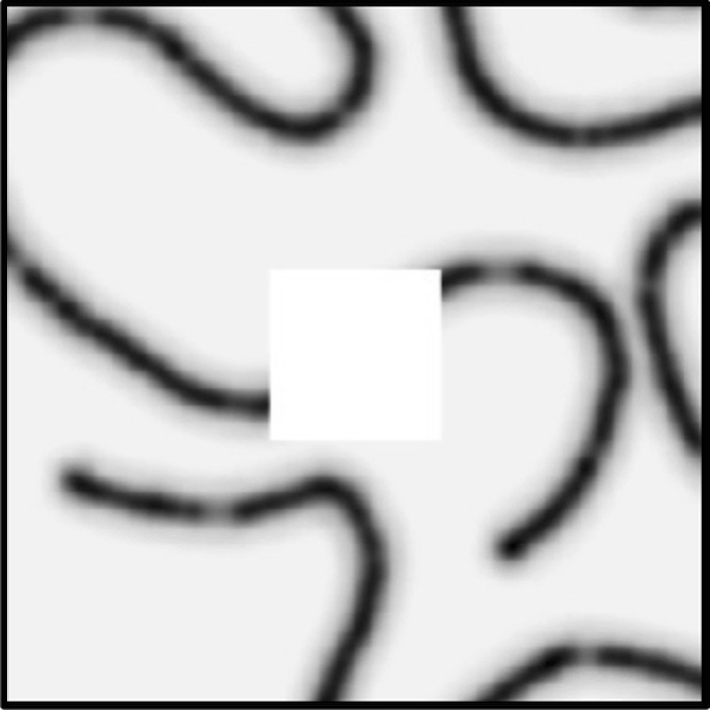
**This figure shows the contents of the Peripheral Priority Map**. The PPM gives the salient locations outside the central attentional field. The locations would be added to the HBPM. Note that higher saliency is represented by darker shading.

**Figure 12:**
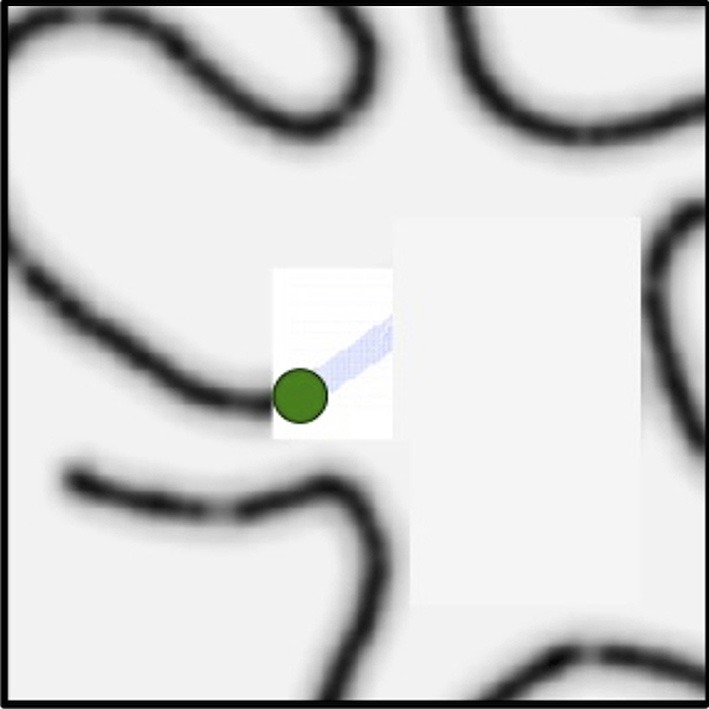
**This shows the History Biased Priority Map**. Once the next central focus (green dot), FHM, and PPM are all combined into the HBPM, this representation can now serve as the basis for selecting the next fixation.

**Figure 13:**
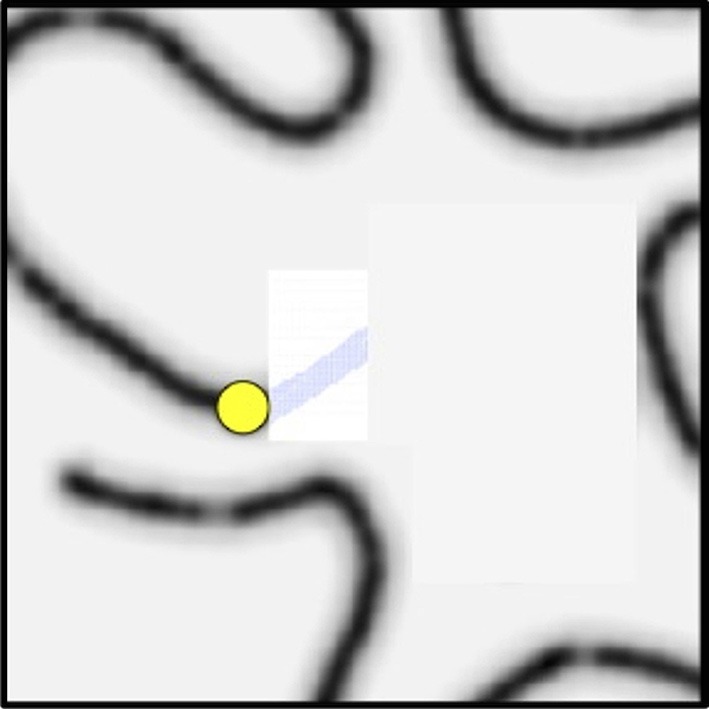
**The HBPM is shown again**. The choice of next fixation is computed from the set of salient peaks with the additional constraint that the next fixation must lie along the curve and be connected to the previous fixation along a portion of the curve not already inhibited. This is the yellow dot and the choice would lead to a saccade.

**Figure 14:**
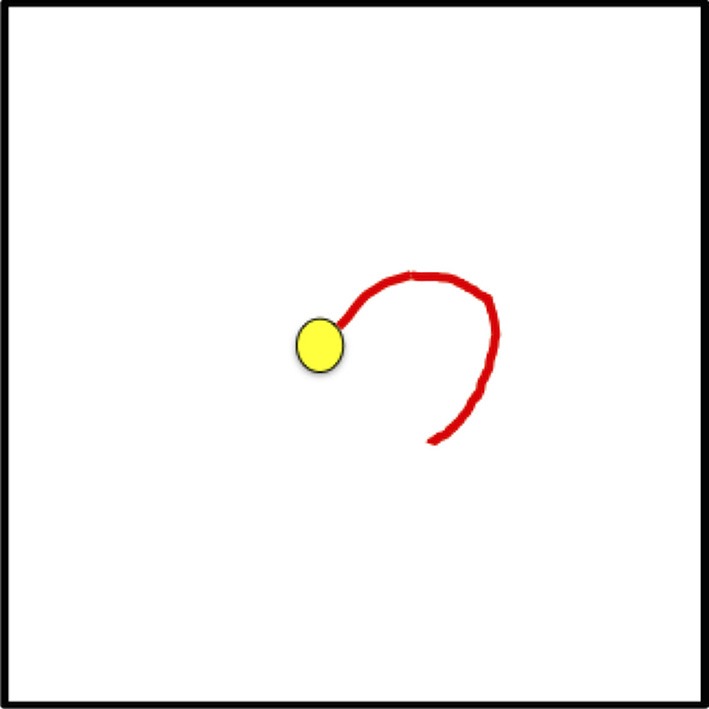
**The FHM is shown, updated to center the new fixation**. Further processing requires the decision to disengage attention from the current fixation, attention to be disengaged while the saccade is executed, the central field moves, the new visual field is processed, the already determined central fixation is attended, the attentional sample is recorded, and the process repeats.

The sequence of figures below (Figures 8–14) show the steps executed by the model in order to achieve a single step of tracing; details are in the figure captions. Clearly, several steps such as these are required to complete the task. Using the CP's of Figure 6, the details required for curve tracing can be seen in the Visual Search: Overt network, although the task specific component of tracing is not specifically shown. Smaller displays might only require the Visual Search: Covert CP.

How does this differ from previous explanations of human curve-tracing behavior? It is a more detailed and generalizable explanation than what was previously presented by Ullman (1984), Jolicoeur et al. (1986) or Roelfsema et al. (2000). Specifically, Roelfsema et al. (2000) propose that attentional mechanisms realize an *attentional label* that spreads along the activated units that belong to the target object or region, binding them into a single representation. Ullman's operations can all be re-interpreted using this spread of the attentional label, either along a curve or over a region. Although this is a sensible proposal it describes the process at one level of representation, that is, it assumes that all required data and computation can be performed within the representation of a curve. How this might generalize to other kinds of visual tasks is an open question. The difference with our approach is that the generalization to a broad set of tasks is more apparent.

## Discussion

In addition to comparisons to the various theories and systems already mentioned, here a further comparison can be made with the Neural Theory of Visual Attention (NTVA) (Bundesen et al., 2005, Kyllingsbæk, 2006). At the outset, the Cognitive Programs that control ST (let's term this as CP-ST for ease of referral) embody major differences when compared to NTVA, both in approach and in goals. NTVA is purely a theory of visual attention, not addressing how vision functions and assuming that the visual system uses the Gestalt principles to segment, in an unspecified manner, the visual scene into objects as part of its first wave of processing. NTVA then describes how objects and features are subsequently selected in the second wave of processing. In contrast, CP-ST attempts to represent the visual process itself, using abstractions of its elements, neurons and synapses, as well as a full set of selection mechanisms. Secondly, the NTVA system relies on the concept of resource allocation at the heart of attentional processing, following many previous works going back to the earliest explanations of attention (Tsotsos et al., 2005b). During the two waves of processing in NTVA, the first allocation of processing resources is at random while in the second pass they are allocated according to attentional weights that are computed for each object in the visual field such that the number of neurons allocated to an object increases with its attentional weight. There is no similar neurons-to-visual-object allocation within CP-ST. The processing architecture is constant throughout processing and only its parameters change that make some neurons more or less selective and connections more or less transmissive. NTVA uses only two mechanisms to accomplish its goals, *filtering* (selection of objects) and *pigeonholing* (selection of features). CP-ST employs many mechanisms in each of the *suppression, selection* (filtering and pigeon-holing are two of ST's 8 selection mechanisms) and *restriction* categories (see Tsotsos, 2011). It is difficult to see how NTVA can account for the top-down latency of attentional modulation, for the attentive suppressive surround, for receptive field narrowing, for inhibition of return, and other aspects of attention as a result while CP-ST inherits these from ST.

A major strength of NTVA is the quantitative comparisons possible using its two major equations, as is illustrated in Bundesen et al., (2005), covering a wide range of effects observed in the firing rates of single cells in primates. CP-ST has yet to prove itself—it is a hypothesis at this stage; however ST recently has shown these same effects (Rothenstein and Tsotsos, 2014) and has the additional strength that it can accept real images and process them, exhibiting attentive behavior as would an experimental subject. There are 10 free parameters for the basic ST equations (Rothenstein and Tsotsos, 2014). The CP-ST framework, however, would have more and at this stage it is unknown what they may be. TVA on the other hand, on which NTVA is based, has a smaller number of parameters, 4 (Andersen and Kyllingsbæk, 2012). Although an ability to represent behavior with as few parameters as possible is an important consideration, it cannot be expected that that complex behavior comes without a price. The trick is to not have more free parameters than needed; it's an Occam's Razor issue. In general, most models are not currently detailed enough for a comparison on this point. Summarizing this comparison, it would be an interesting and likely valuable research project to detail the connections between NTVA and CP-ST and to see if unification might lead to a productive result.

The Cognitive Programs framework, although containing elements seen in other models, provides an implementable (see Kotseruba and Tsotsos, 2014) model that we hypothesize exhibits task behavior comparable to the behavior of human subjects performing the same tasks.

## Conclusions

**Figure 15:**
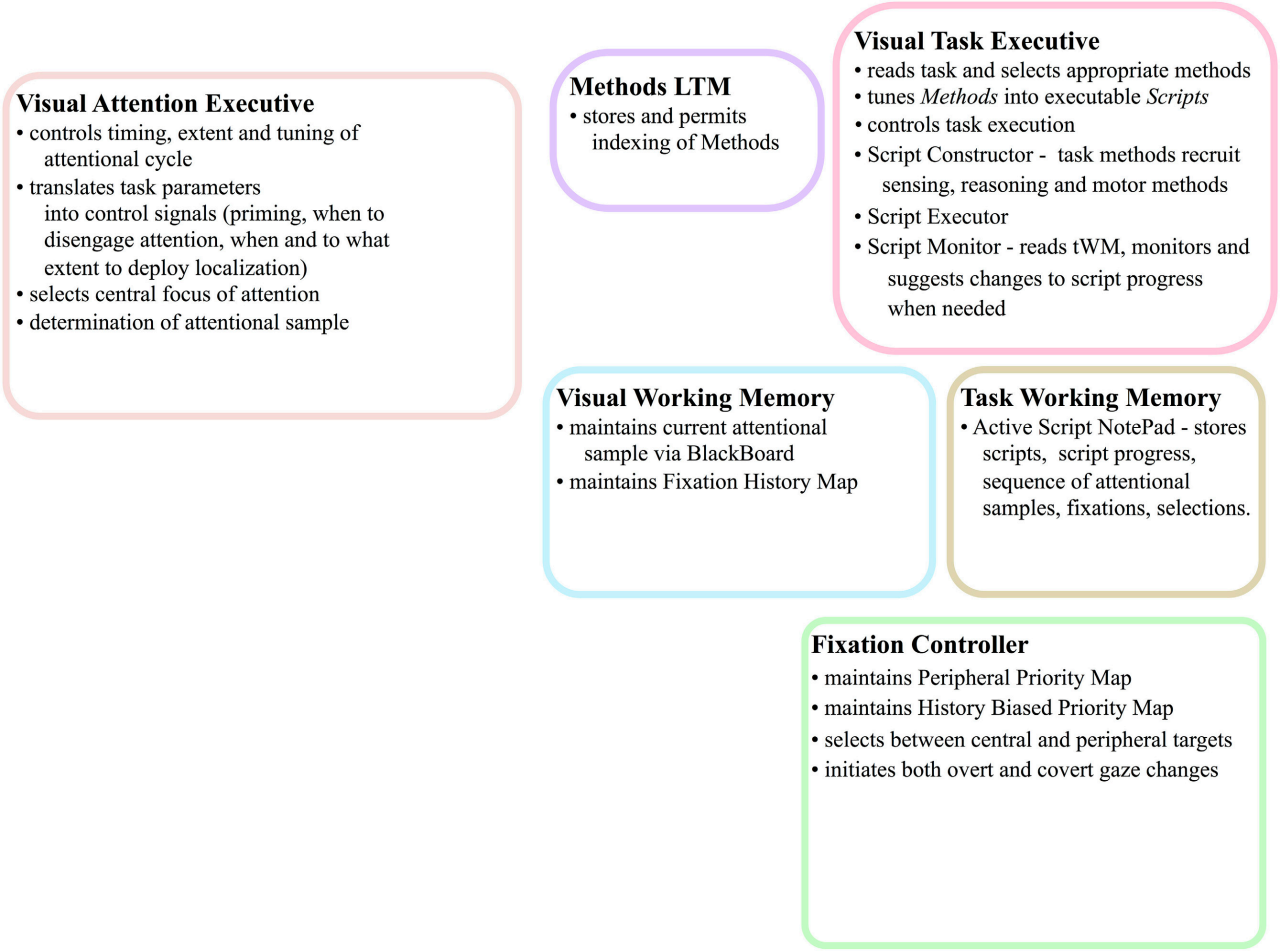
**The abstract, block-diagram structure of the functional components of Figure 5 is shown, this time with summaries of the main tasks that each must perform**.

This paper began with a question: What are the computational tasks that an executive controller for visual attention must solve? The answer is not a simple enumeration of tasks as one might have hoped. Rather, exploring this question has led to a complex set of inter-connected and cooperating functional components, each a hypothesis with several sub-hypotheses within. Figure 15 summarizes the tasks that our controller must address—in other words, this is the answer to our original motivating question—using the same figure structure as Figures 5, 6. Within each box, the major tasks that must be performed are listed and these arise from the detailed structure of Figure 6.

The value of a hypothesis rests solely with the possibility of testing its validity and here it is important to ensure the proposed components can be tested. Testing would proceed computationally to ensure computational performance with respect to human behavior is satisfactory as well as experimentally in search of evidence supporting the many predictions of the overall theory. The task would be daunting if the framework of Figure 6 was composed of entirely new components. However, the fact that so many components have already been examined with success gives the hypothesis represented by the overall integration some degree of plausibility.

The full system of Figure 6 can be best tested via computational implementation. A success would provide an existence proof that all of these components perform their intended function and that in concert they function as a controller for visual task execution. Such a test is not easy to conduct but it is important that any test use images and a non-trivial task. This has been accomplished to a large degree through a computer system that plays a video game (Kotseruba and Tsotsos, 2014). This implementation did not test all of the elements of Figure 6 but does test the subset required for the game and also demonstrates that the form of the CP's presented in Figures 1, 7 is feasible.

With respect to the CP's shown in Figure 7, it is important to note that all of these represent computationally confirmed processes. That is, they are simply encodings of the algorithms presented in our past publications. In some cases elements are also supported by experiment. For example, the Localization function includes sub-components of an attentive suppressive surround and also requires this to be a result of recurrent processes. These two elements have experimental support (Hopf et al., 2006; Boehler et al., 2009). However, the extraction of the attentional sample from the Localization function and its use within the BB of the CP framework does not yet have experimental support. The Visual Search CP's generally encode behavior that is well documented experimentally in the visual search literature and also is shown to be a characteristic of ST (Rodríguez-Sánchez et al., 2007; Tsotsos, 2011). Finally, the VH of Figure 4 is also defined and shown to perform under attentive conditions (Tsotsos et al., 1995, 2005a; Tsotsos, 2011; Rodríguez-Sánchez and Tsotsos, 2012; Rothenstein and Tsotsos, 2014). The fixation control (FC) component has also been implemented and successfully tested (Tsotsos et al., 1995; Zaharescu et al., 2005; Bruce and Tsotsos, 2009; Wloka, 2012) with all of its sub-components included. Attentive behavior and predictions of Selective Tuning has been extensively tested both with computational and human experiments (detailed in Tsotsos, 2011; Rothenstein and Tsotsos, 2014).

Cognitive Programs grew out of Ullman's Visual Routines, but represent a generalized and updated conceptualization. Although many aspects of the CP framework have been successfully tested, some computationally and some experimentally, the full framework awaits testing as do the many experimental predictions that it expresses. The main hypothesis presented by this paper then is that the Cognitive Programs framework, built upon the substrate of the Selective Tuning model, suffices to provide an executive controller for ST, and that it also offers a testable, conceptual structure for how visual task execution might be accomplished.

### Conflict of interest statement

The authors declare that the research was conducted in the absence of any commercial or financial relationships that could be construed as a potential conflict of interest.

## Footnotes

^1^This process was first described in Tsotsos (1991), and is fully detailed in Tsotsos (2011) and Rothenstein and Tsotsos (2014). Its mathematical properties, under the assumptions of the overall model, plus a strategy for monitoring and correcting the top-down traversal are also detailed in those presentations.
